# The aporetic dialogues of Modena on gender differences: Is it all about testosterone? Episode II: Empathy

**DOI:** 10.1111/andr.70037

**Published:** 2025-04-12

**Authors:** Giulia Brigante, Clara Lazzaretti, Ali Ahmad, Massimiliano Colzani, Filippo Vignali, Michele Zoli, Manuela Simoni

**Affiliations:** ^1^ Unit of Endocrinology, Department of Biomedical Metabolic and Neural Sciences University of Modena and Reggio Emilia Modena Italy; ^2^ Unit of Endocrinology Department of Medical Specialties Azienda Ospedaliero‐Universitaria of Modena Modena Italy; ^3^ Department of Biomedical Metabolic and Neural Sciences Center for Neuroscience and Neurotechnology University of Modena and Reggio Emilia Modena Italy

**Keywords:** behavior, emotion, empathy, gender, sex, testosterone

## Abstract

The exploration of gender differences in non‐andrological fields was the core focus of a series of discussions, which took place at the Endocrinology Unit in Modena, Italy in the form of the aporetic dialogue of ancient Greece. This second episode reports the transcript of the actual debate on testosterone's role in defining empathic behavior in males and females. The two groups of discussants sustained that empathic gender differences may rely either on testosterone exposure (group 1) or on other factors (group 2). The first group supported the hypothesis that females are more empathic than males due to reduced exposure to fetal testosterone, which correlates with higher empathic scores at all ages and lower sensitivity to testosterone in adulthood. This hypothesis is also supported by evolutionary mechanisms and evidence in animal ethology. Conversely, the second group affirmed that gender differences rely on structural diversities in brain organization, hormonal factors such as vasopressin, oxytocin, and cortisol, as well as sociological aspects. An expert in neurophysiology, acting as a referee, moderated the discussion and decided whether the two theories were equivalent or one was predominant.

## 1 INTRODUCTION

This is the second paper in a series dedicated to exploring the role of testosterone in determining gender differences in various fields. In the first article published in the Andrology and Humanities series, we examined the role of testosterone in influencing the different criminal attitude in males compared to females.^1^ In this episode, we wonder if testosterone plays a role in determining different empathic responses according to gender.

## 2 METHODOLOGY

The discussion was carried out according to the scheme of the Socratic aporetic dialogue: the question of the definition or explanation of a phenomenon is posed and then discussed, without necessarily reaching a truth. Aporetic dialogues typically end in *aporia*, a state of puzzlement by way of the equality of opposite reasonings. The discussants could discover that the opposing reasons only apparently balance each other and therefore identify the most promising theory, or that the two theses are actually equivalent, so the investigation must take a new turn or stop.

Discussants were subdivided into group 1 in favor of the existence of a gender difference and supporting a prominent testosterone role, and group 2 which claimed that factors other than testosterone were responsible. The dialogue took place as a seminar event open to the public.

Filippo Vignali (FV) explained the meaning of the term empathy, the techniques for measuring it and the evident gender differences in animals and in humans (group 1). Then, Ali Ahmad (AA) illustrated gender‐related differences in neural substrates involved in empathy (group 2). Clara Lazzaretti (CL) tried to demonstrate that the dimorphic empathic attitude in males and females is due to testosterone serum levels (group 1). Finally, Massimiliano Colzani (MC) argued that these gender differences are due to other hormonal factors besides testosterone (group 2).

Michele Zoli (MZ), neurophysiologist, had the role of commenting on the data and, in the end, deciding whether one of the two theories was successful or whether the two were equivalent (referee).

The discussion was open to questions or comments from the audience composed of endocrinologists and resident doctors, biologists, biotechnologists from the Endocrinology Unit of Modena, physicians in specialist training from the School of Forensic Medicine, doctors in specialist training from the School of Psychiatry (audience).

The organization of the dialogue and the collection of the data presented was managed and supervised by Giulia Brigante (GB), based on an idea by Manuela Simoni (MS).

## 3 THE APORETIC DIALOGUE

**FV (group 1)**: The best way to explain empathy is starting from etymology. The term derives from the ancient Greek “εμπάθεια” (empátheia), a word composed of en‐(inside) and ‐pathos (suffering or feeling). In ancient theatrical language, it symbolized the emotional bond that tied the bard to the audience. However, the modern meaning of the word empathy has a different origin. It actually derives from the German “Einfühlung,” a term that the philosopher and art historian Robert Vischer conceived in 1873 as the ability to feel within and/or to feel with, to perceive the external nature as belonging to our own body, the ability of the human imagination to grasp the symbolic value of nature.^2^

If I ask you to tell me what empathy is, I bet there will be similar answers but with different nuances. I did my best to select a complete definition: empathy is the ability to understand and share the internal states of others; it is a complex, multidimensional phenomenon that includes several functional processes, such as emotion recognition, emotional contagion, and emotion priming, as well as the abilities to react to the internal states of others, and to distinguish between one's own and others’ internal states.^3^

Now, to demonstrate that gender difference exists, I will start with animal studies, which demonstrate empathic gender differences in some species of mammals and birds. Langford et al.^4^ studied mice's responses when watching a conspecific in pain, with the pain‐associated behaviors in the viewer possibly indicating the level of empathy. They created a platform with the studied mouse placed in the center, with two transparent cages on both sides so that the central mouse could see what was happening on its sides. One cage contained a healthy mouse, and the other a mouse subjected to an intraperitoneal injection of acetic acid, resulting in observable pain behavior. The researchers calculated the “location scoring” of the studied mouse, showing that female mice were on average closer to the cage where the injected mouse was located, whereas male mice showed no preferences. The authors concluded that being closer to the aching mouse could reflect an emotional proximity to the similar individual.

**MS**: In which phase of the estrous cycle were the female mice?

**FV (group 1)**: The authors did not specify it.

In a similar study, female rats were found to have lower latency of cage opening and higher average of attempts to release a fellow in distress compared to males.^5^

In Eurasian jackdaws (*Corvus monedula*), a particular species of corvids, empathy was evaluated through a series of elaborate experiments, in which individuals could express an altruistic choice—sharing food with the conspecific—or a prosocial one—equally dividing the available food, or a selfish one—keeping the food for themselves.^6^ The result was that females exhibited more altruistic and prosocial choices compared to males.

Also in chimpanzees, females are more likely to exhibit pro‐consolation behaviors, such as embracing and caressing, toward companions mistreated by other individuals.^7^ In the literature, apart from great apes and humans, the only mammals described to perceive higher forms of empathy, such as cognitive empathy and altruism, are dolphins and elephants, which are species with a social organization and, in case of the elephants, with a matriarchal structure.^8^ Nevertheless, to date, there are no studies investigating the gender differences in those populations in terms of empathic attitude. A deeper knowledge of gender empathy perception in invertebrate and vertebrate matriarchal species would support the hypothesis that pro‐social/empathic behaviors have common ontological and phylogenetic roots rather than a social and cultural origin.

But does this gender difference persist in humans? First, we tried to identify social indicators of empathy. We looked at data published by ISTAT (*Istituto Nazionale di Statistica*, the Italian National Institute of Statistics), ISS (*Istituto Superiore di Sanità*, the Italian National Institute of Health) and AGENAS (*Agenzia Nazionale per i Servizi Sanitari Regionali*, the National Agency for Regional Health Services). In Italy, women are more likely to donate organs^9^ and engage in charity works.^10^ Women predominantly head single‐parent families, while men prefer to live alone.^11^ We then considered “empathetic” occupations: about 70% of National Health Service employees and 75% of caregivers are women.^12^ The only findings in favor of men were that they are more likely to be members of volunteer associations and to donate blood.^13^, ^14^ However, these are only surrogates for empathy that might be influenced by cultural aspects linked to a patriarchal society, which sees women as more inclined to caregiving jobs than men.^3^, ^15^ Let us see how empathy is scientifically measured in human beings and if such measurements reveal a gender difference.

The first test we will consider is the interpersonal reactivity index (IRI), a questionnaire that assesses self‐perceived empathy.^16^ An Italian study, demonstrated through the IRI test on approximately 300 young subjects of both sexes aged 18–31 years that women perceive themselves as more empathetic than men.^17^

Another test widely used to assess the degree of empathy is the “Reading the Mind in the Eyes” (RMET).^18^ It includes 36 photographs of male and female eyes depicting emotional states. For each picture, the participant is asked to choose the emotional state that best describes the eyes, selecting one out of four possible emotions. In the following study, authors tested sex and age differences using the English version of the RMET in adolescents and adults across 57 countries. On average, females show higher accuracy in their responses and with shorter response latency compared to males.^19^

Finally, the point light display (PLD) test is a method that involves the placement of LED lights on the body of an actor, who is asked to perform movements that mimic an emotion. In this emblematic study, participants were asked to recognize movement, gender, and the underlying emotion conveyed by the actor's movement inside a dark room. Once again, women showed greater accuracy than men and shorter response latency in perceiving the actor's action and emotion.^20^ Moreover, when LEDs were placed on an actor's face who portrayed an emotion, females had a faster response time and a better ability to recognize faces conveying sad or neutral emotions.^21^ Conversely, males easily recognize happy female faces, a skill that, according to the theory of sexual selection, would enable the recognition of a partner inclined to mate.^21^

Summarizing, several studies demonstrate that there are gender differences in empathy, supporting that females are more empathic both in animals and humans.

**MZ (referee)**: I have a question regarding the study in which RMET was applied in adolescents and adults across 57 countries.^19^ Were there any differences or similarities among countries?

**FV (group 1)**: The female advantage in RMET performance is evident in almost all countries. Interestingly, it is negatively linked to prosperity and autonomy, and positively correlated with the collectivism of the country.^19^

**Audience**: It would be even more interesting to evaluate empathy in animal groups with a strong hierarchical organization. In this context, the empathic power of females stands out, as they could use it to “make themselves accepted” or to “get what they want”. Vice versa males do not need to be empathetic maybe because they impose their will in other ways.

**GB**: I just want to underline why talking about gender differences in empathy makes sense. Clarifying these mechanisms is important to understand what happens when they become dysfunctional. First, there is a striking gender difference in autism, a disease characterized by dysregulation at various levels of empathic abilities: the prevalence of this condition in males is significantly higher than in females.^22^ Additionally, the economic sector, from marketing to finance, is extremely interested in studying these dynamics, to personalize the offer on a gender basis. Advertising is targeted differently if the product is addressed to females rather than males: the product's packaging or appearance on social media algorithms changes accordingly. To evaluate empathic gender differences in economics, a test called “the ultimatum game” exists, and it is also used in biological and neuroscientific fields to measure the degree of empathy by recreating a specific situation: the subject must decide whether to keep the money or share it, somewhat akin to the earlier bird deciding whether to keep the seeds or share them.^23^ In summary, the study of these gender differences is not a mere intellectual exercise but has important practical implications in various fields.

**AA (group 2)**: I will try to delve a bit more into detail about the various components of empathy and the gender‐related differences in neuroscientific substrates.

For that purpose, I want to show you a picture. I ask you to look at it in silence for a few seconds and focus on possible emotions and thoughts that this image may evoke in you.

**FIGURE 1:**
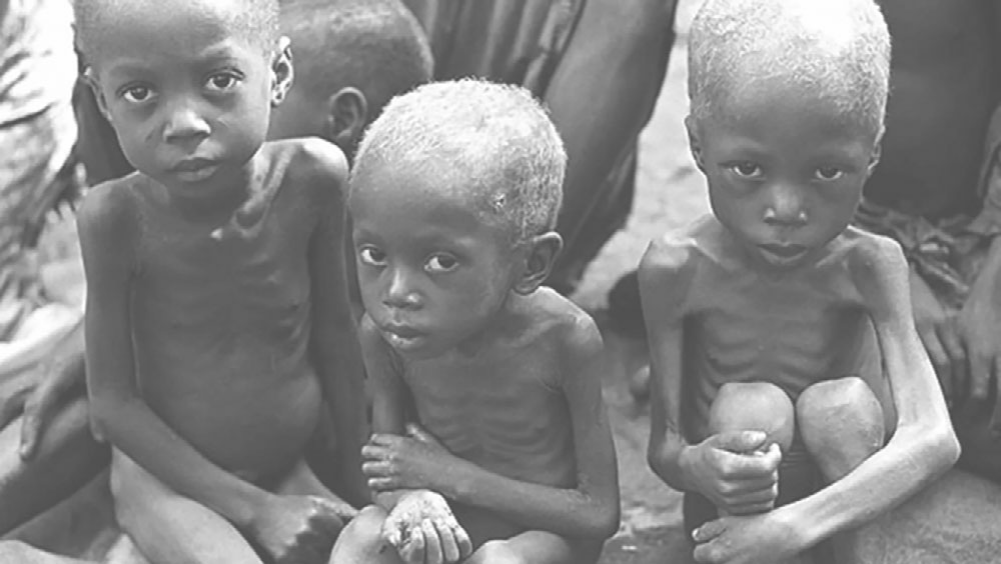
“Each day 25,000 people, including more than 10,000 children, die from hunger and related causes. United Nations.”

*AA shows a picture of three young black children, seated on the ground, who appear to be suffering from severe malnutrition with their ribs and bones being visibly prominent, indicating extreme hunger. The children have somber expressions, reflecting a sense of hardship and suffering. In the foreground, there is a caption that reads: “Each day 25,000 people, including more than 10,000 children, die from hunger and related causes. United Nations.”* (Figure 1).

Each of us reacted depending on the level of attention, emotional experiences, knowledge of the topic, and sensitivity toward it. However, your empathic reactions can be summarized as emotional reactions, such as feeling sad, distressed, unpleasant, negatively overwhelmed, angry, or cognitive reactions, such as thinking about children's mental state and emotions, imagining how you would feel if you were in their place.

These two types of responses reflect the two components of empathy. The first represents affective or emotional empathy that stems from the emotional state or condition of another and is congruent with the other person's emotional state or situation.^24^ This phenomenon, also called “mirroring,” manifests at a preconscious level and is present from the earliest days of life.^25^ The second type of reaction, namely cognitive empathy, is an intellectual process defined as the ability to understand another's perspective or mental state. In simple terms, it means putting oneself in someone else's shoes. As you grow, the cognitive component becomes increasingly important. It is also referred to as perspective‐taking or mentalizing.^25^, ^26^ Gender differences are more evident in the affective component,^3^ thus I will focus on this kind of empathy.

The neuroscientific models describe affective empathy (e.g., embodied simulation theory or perception‐action model) as a shared emotional experience between individuals who simulate the mental and emotional states of others in their minds, allowing them to understand and resonate with their experiences at a preconscious level. These models suggest the involuntary recruitment of common neural substrates in self‐related and other‐related emotional states.^27^, ^28^

Electrophysiological studies may confirm this thesis suggesting the activation of a common affective and sensory‐moto resonance system between individuals, may explain other's emotion‐understanding. For example, a study by Swedish psychologists,^29^ which measured the electrical activity in facial muscles linked to specific expressions, showed that even without conscious awareness, people's facial muscles react differently to happy and angry faces. Similar evidence was demonstrated for painful stimuli.^30^

The neural substrates underlying this phenomenon rely in part on the mirror neurons system.^31^, ^32^ Mirror neurons were discovered in the 1990s by a group of Italian neuroscientists while conducting experiments on the motor system of macaque monkeys.^33^ They activate either when an animal acts or observes the same action performed by someone else. Interestingly, these neurons have multimodal activation as they respond both to visual and associated sensory information, such as auditory or tactile cues related to those actions. Following electrophysiology and functional imaging experiments have demonstrated their presence in humans as well.^34^ These neurons are believed to play a crucial role in learning but even more importantly in understanding the actions and emotions of others.^26^, ^31^, ^32^

Subsequent electrophysiological and imaging studies have provided significant evidence regarding mirror neurons' involvement in the execution of empathic tasks. A meta‐analysis analyzed data from several functional magnetic resonance imaging (fMRI) studies conducted while subjects were directly exposed to painful stimuli or watching others being exposed to the same stimuli.^35^ The anterior insula and the anterior cingulate cortex, which are connected to the limbic system and responsible for the representation of emotional and affective states, as well as the inferior frontal gyrus and the inferior parietal lobe, which are thought to be parts of the human mirror neuron system, emerged to activate in both subject groups.^31^, ^36^

Now, I would like to bring to you a couple of studies focusing on the gender differences observed in the mirror neuron system.

Cheng and colleagues^37^ investigated the sex differences in the neuroanatomy of the human mirror‐neuron system (hMNS) using voxel‐based morphometry,^38^ a technique widely used to investigate brain anatomy (e.g., volume of gray matter, white matter, or cerebrospinal fluid) by applying statistical analysis on high‐resolution structural MRI images. The investigators found that gray matter volume in the pars opercularis and inferior parietal lobule—integral parts of the hMNS—is significantly larger in females and this correlates with higher self‐reported scores in the emotional empathic skills. In another study,^39^ functional MRI was performed on enrolled participants while they were shown faces expressing emotions (fear or anger). During these empathy tasks, they were asked to focus on their own emotional response (self‐task) or to evaluate the emotional state expressed by the face (other‐task). Additionally, they filled out surveys on empathic abilities. The results showed that the self‐ and the other‐task activated different regions including the inferior frontal gyrus bilaterally, implicated in the hMNS, and activation was stronger in females than males. Moreover, the activity in these regions was associated with dispositional empathic traits and self‐reported emotional experience during the experiment, indicating a significant connection between the level of interpersonal emotional engagement and mirror neuron activation.

I conclude by reiterating that gender‐related emotional empathy differences could be explained, at least partly, by differences in volume and function of human mirror neuron network in the two sexes.

**Audience**: I was very impressed by a graph shown by AA which displayed empathic response latency times concerning a stimulus produced by a person's angry, neutral, or happy face, where the angry face induced a much‐delayed response or no response at all.^29^ This reaction is probably intentional, as the individual may choose to avoid confrontation. Based on this, I wonder how suitable the tests used are for assessing gender differences, where perhaps a woman performs better in certain contexts or tests, while the same test is not appropriate to reveal male empathy, which may perform better in other contexts.

**AA (group 2)**: Thank you for the comment because you have grasped an interesting point. Tests which evaluate and score empathy are subjected to limitations, which might be linked to the age of tested subjects,^40^ or their intentionality.^15^ Indeed, a gender gap expectation exists, as it seems that women tend to perform better because of the social expectations and stereotypes placed upon them. Society indeed associates women with the role of caregiving and nurturing, which they feel obligated to fulfill.^3^ Furthermore, it has been observed that their performance varied depending on the contexts in which women were tested.^15^

**CL (group 1)**: The data shown by AA on neuroanatomical differences are certainly interesting, but I think that the main role is played by circulating levels of testosterone, as *primum movens*. Most of the studies in the literature report higher empathic performance in females than in males.^3^ In fact, both functional and structural differences are found in female‐ and male‐specific brain areas, such as the hypothalamus, limbic system, and neocortex, which are regions activated during the emotional involvement of the individual. These same areas are demonstrated to have high‐expression levels of androgen receptors, underlining their responsiveness to these steroids.^41^, ^42^, ^43^, ^44^ Studies conducted in animal models indicate that testosterone plays a crucial role in the modulation and regulation of the development of specific brain structures.^45^, ^46^ This suggests that testosterone intervenes during prenatal development, determining differences between the two sexes. In humans, many studies demonstrate a negative correlation between the level of exposure to fetal testosterone and social behaviors.^3^ Among these, the Cambridge Study is one of the most important.^44^, ^47^, ^48^, ^49^ This longitudinal study, initiated in the 1990s, followed a cohort of 183 children (100 boys and 83 girls) from fetal development to 6/9 years old. It aimed to correlate fetal testosterone exposure with the developmental stages of empathic behavior in children. Thus, testosterone levels in amniotic fluids were measured during pregnancies, and at different ages, children were tested for several empathizing tasks. Children were tested for eye contact at 12 months,^49^ for vocabulary range at 24 months,^48^ and for the quality of social relationships and the number of interests at 48 months.^47^ Afterward, their empathizing quotient was measured at 6/9 years through a questionnaire and the completion of the child versions of the RMET.^44^ For each of these parameters, girls always achieved higher scores than males and negatively correlated with fetal testosterone.

As a further confirmation that androgens have a role in the development of empathizing behavior, a study enrolled 75 pregnant women, 30 of whom were affected by hyperandrogenic polycystic ovary syndrome (PCOS), and their amniotic testosterone level was measured. Empathy quotient and systemizing quotient tests were administered to children's parents to assess trends in gender‐typical behavior at the age of 4–11 years. While no statistical difference was found between boys born from healthy or PCOS women, the empathy and systemizing quotients of girls born from PCOS women, who had been exposed to higher levels of fetal testosterone, resulted significantly lower and higher respectively compared to girls born from healthy women.^50^

Exposure to high prenatal testosterone levels seems to affect empathy attitude also in adulthood.^51^, ^52^, ^53^ Fetal testosterone exposure can be estimated through the second to fourth digit ratio (2D:4D ratio), which is lower in case of higher exposure. In the study by Van Honk, 16 young healthy women were enrolled and tested for the RMET.^53^ Subsequently, part of them were administered sublingual testosterone and the remaining were treated with placebo, followed by the repetition of the test. The authors observed that women treated with testosterone performed less than the placebo group, demonstrating an acute activity of testosterone in modulating empathic behavior. Interestingly, women with a lower 2D:4D ratio, and thus who had been exposed to higher levels of fetal testosterone, presented a more evident negative effect of testosterone on test performance and their empathic attitude.^53^ Therefore, fetal testosterone exposure not only influences the prenatal development of specific brain areas but also predispose to a higher testosterone susceptibility in adults.^51^, ^52^, ^53^, ^54^, ^55^, ^56^ The same research team investigated which brain areas were affected after testosterone injection through fMRI. They observed that the testosterone treatment altered the connectivity between areas involved in the integration of sensory information or that are activated to produce a response to emotions.^52^

Another condition that demonstrates androgens’ effect on empathy in adults is anabolic androgenic steroid dependence. A study conducted in Norway collected data from patients of 38 substance use disorder treatment facilities, demonstrating that subjects who had high symptoms of anabolic androgenic steroid dependence showed more aggressive and impulsive personality traits as well as reduced empathy quotients compared to patients with lower symptoms of dependence.^57^

Congenital adrenal hyperplasia (CAH) is a pathological condition that causes hyperandrogenism and can be exploited to evaluate androgens’ role in empathic behavior. Unfortunately, few papers directly evaluate the empathic quotient in these patients; however, there is evidence that females affected by CAH show increased male‐typical interests and game preferences and lower caregiving attitudes and scores related to empathy.^46^, ^58^

I hope I have convinced you that testosterone has a fundamental role in modulating human empathic behavior.

**MC (group 2)**: Ok, CL you have convinced us that testosterone plays a role, but I think it is not the only hormone responsible for the gender difference in empathic aptitude. Other hormones can have a role, and I want to start with vasopressin. It is primarily associated with water balance and blood pressure control. Moreover, it is a neurohormone that centrally modulates the centers responsible for behavior and emotions, such as aggressiveness, psychosocial stress, empathy, and altruism.^59^ Interestingly, this neurohormone has different actions in males and females. Vasopressin in females promotes friendly behaviors and attitudes and social reinforcement, while in males it modulates aggressiveness only toward other males and reinforces bonds with partners, offspring, and individuals considered part of the same group.^59^, ^60^, ^61^, ^62^ It has been observed that the administration of vasopressin in men reduces emotion recognition when observing male gazes, but not when observing female gazes, and this reduced recognition of emotions is greater for negative emotions than for positive ones. However, this phenomenon was not observed in females.^60^, ^62^ In another study, intranasal vasopressin was administered to a sample of males and females and their involuntary response of facial muscles to neutral, happy, and angry expressions of people of the same sex was evaluated. They observed that the administration of vasopressin in males induced the contraction of the corrugator muscles of the forehead, making them appear more angry/aggressive and less approachable. On the contrary, in females vasopressin induced the contraction of the zygomatic muscles, thus promoting a smiling expression and a more friendly appearance.^60^ Why such a gender difference? Vasopressin is a stress hormone, and its secretion increases in stressful situations, so it seems that the different action in males and females can be attributed to the different survival strategies adopted by the two sexes.^60^

Another neurohormone that plays an important role in the modulation of emotions and behavior is oxytocin,^59^, ^61^ which is produced during labor to induce uterine contractions, but also to strengthen the bond between child and parents, both the mother and the father.^63^, ^64^ However, I failed to find gender differences in this action.

Another hormone acting in the central nervous system in the modulation of behavior, emotions, and empathy is cortisol.^65^, ^66^ In many studies, it has been observed that high levels of testosterone lead to a greater tendency toward aggression, dominance, and reduced empathy, but the effect of testosterone was secondary to cortisol levels. This theory is called the dual‐hormone hypothesis.^65^ In the specific case of empathy, elevated cortisol levels improve empathic faculties even when testosterone was at high levels. In a study conducted on a sample of university students, both males and females, empathy was assessed using the RMET and the IRI (eyes test), which evaluates both affective and cognitive empathy. In these subjects, salivary testosterone and cortisol levels were measured in the morning before performing the tests. Compared to males, females had higher scores on both tests. Furthermore, no correlation was observed between testosterone, cortisol levels, and empathy in females, but as cortisol increased, empathy increased as well. On the other hand, high testosterone levels in males were associated with lower empathy, but only in the presence of low cortisol.^66^ The impact of cortisol on empathic behavior is supposed to be due to its inhibitory effect on the hypothalamic–pituitary–gonadal axis and the androgen receptor expression, antagonizing testosterone action on aggressive and dominance attitude.^66^, ^67^, ^68^, ^69^ The idea that high cortisol levels may correlate with increased empathy is in line with other studies demonstrating that high testosterone can correlate with altruistic behaviors. For example, it has been observed that in intergroup competition, males with higher testosterone, and simultaneously elevated cortisol, tended to be more competitive against members of the other group but to cooperate more and exhibit prosocial behaviors toward members of their group.^70^ The action of cortisol in modulating testosterone is not well understood but it seems that it is more useful to reduce the tendency toward dominance and promote collaboration to overcome stressful situations.

I also explored any possible role of female hormones, such as estrogens and progesterone, on empathic abilities. However, I could not find data about the correlation between estrogens and empathy in men. In women, it seems that estrogens may play a role in making them more empathic.^71^ A study evaluated the variation in empathy in women during the menstrual cycle. Women in the early follicular phase, when both estradiol and progesterone are low, have greater accuracy in recognizing others’ emotions, especially the negative ones, than those in the mid‐luteal phase, when sex steroids are elevated. Particularly, the increase of progesterone levels in the mid‐luteal phase reduces the reaction time in recognizing negative emotions and the ability to assign the correct emotion to facial expressions. However, no difference was observed in the ability to empathize with others’ emotions between the two phases of the cycle independently from estradiol and progesterone levels.^71^

Finally, to go beyond hormones, I want to show you this study where the difference in empathy based on gender and sexual orientation was assessed. The authors found that regardless of gender, it was the gender the subjects were attracted to, to determine the level of empathy: subjects attracted to men resulted more empathetic than subjects attracted to women.^72^

**FIGURE 2:**
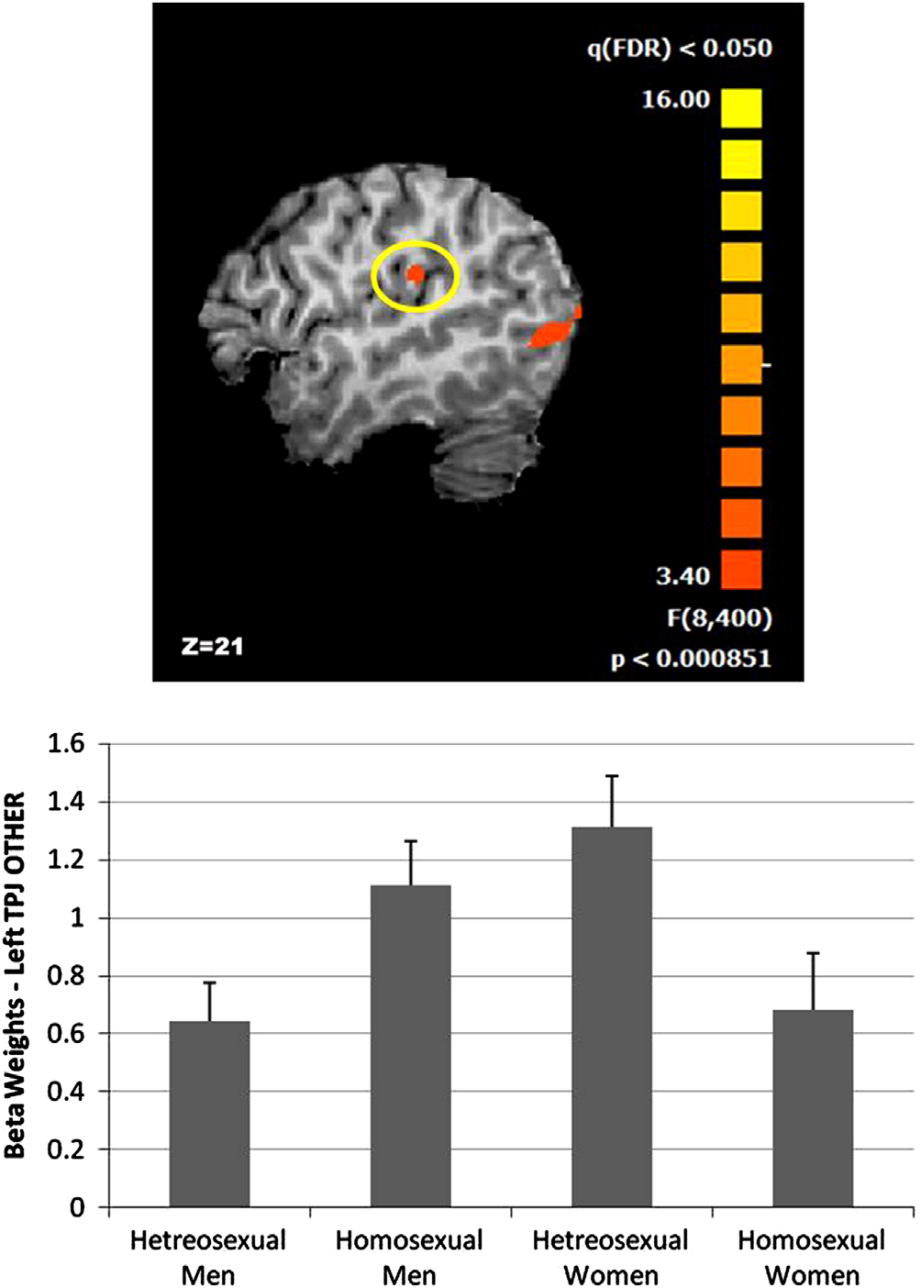
(A) Left TPJ region (yellow circle). (B) Mean left TPJ beta weights activation for conditions referred to others across sexual orientation groups.^72^ TPJ, temporo‐parietal junction.

Empathy was analyzed by measuring the activation of the left temporo‐parietal junction (TPJ) through MRI, while the subjects were evaluating a fact regarding another individual. The left TPJ is a brain area involved in various social behaviors, including empathic processing, self‐referential thought formulation, mentalizing others, and emotional evaluation. In this graph (Figure 2), you can see that during the mentalization of situations involving another individual, this area is more activated in heterosexual women, to slightly lower levels in homosexual men, and to lower and similar levels in heterosexual men and homosexual women.^72^

In summary, gender differences in empathy are not completely explained by different testosterone levels alone because: (a) there are other neurohormones involved in empathy modulation that have different effects depending on gender, such as vasopressin; (b) testosterone action in modulating empathy and social behavior seems to be subordinated to cortisol activity; (c) female hormones, such as estrogen and progesterone, also play a role in modulating empathy in females; (d) the activity of brain areas that mediate empathic thinking does not always correlate with levels of circulating sex hormones but depends on pre‐existing structural characteristics that are not easily attributable to these hormones.

**MS**: Do you know if hypogonadal men are more empathic than normogonadic men?

**MC (group 2)**: A study performed on patients affected by Klinefelter syndrome demonstrated lower empathic quotient scores despite lower levels of testosterone.^73^ However, I must point out that Klinefelter syndrome patients also have significantly lower levels of estradiol compared to women.

**Audience**: I think that the comparison between hypogonadal and elderly subjects should be interesting. Studies by Andrew A. Dwyer on patients suffering from Kallmann syndrome, a form of hypogonadotropic hypogonadism, demonstrate that congenital hypogonadism can be associated with reduced emotionality; however, empathy is not specifically assessed.^74^ In these patients, testosterone replacement does not improve the reduced emotionality, suggesting that it may be due to consequences of the delayed diagnosis or difficulties during pubertal development.^74^ Conversely, there is a lack of data on emotionality in women suffering from hypogonadotropic hypogonadism.

**MZ (referee)**: You focused your discussion on affective empathy. However, we cannot exclude the possibility of different emotional and cognitive responses in men and women. If we consider cognitive empathy, the differences between sexes are much more nuanced, while the evidence is much stronger for the automatic aspect. Moreover, could there be any age effect on empathic behavior? It is widely experienced that emotionality and empathy increase significantly with age. Could this be due to the decline of testosterone levels in the elderly?

**GB**: Although our research has not focused on studies conducted on the elderly, we have not encountered references to advanced age. Most studies were conducted on newborns or children to demonstrate that observed gender differences are not dependent on social and cultural diversities.^75^, ^76^ Additionally, we have not yet mentioned that the common idea is that this empathy gender difference is due to females’ involvement in offspring caregiving, and many articles tend to support this hypothesis.^3^, ^77^, ^78^

**FV (group 1)**: In the study, we previously mentioned regarding the analysis of performance on the RMET in 57 countries, individuals between 18 and 70 years were enrolled, and women consistently scored higher than men, while performance decreased for both sexes as age increased.^19^

**MZ (referee)**: Still, this kind of test evaluates automatic responses but hardly assesses affective involvement, which does not necessarily correspond with the recognition of emotions. Furthermore, performance on this test may be biased as cognitive abilities typically decline in the elderly.

**Audience**: I have a comment about the methodological limitations that various studies on empathy may have. We assume that steroids can modulate brain functioning and organization by regulating its morphological formation. However, this information is often affected by methodological and technical limitations; indeed, it is difficult to understand retrospectively what happened to the fetus when it was in the maternal womb. Therefore, many technical and methodological aspects greatly influence these studies, which rely on indices that evaluate indirectly androgen exposure in fetal life, such as the 2nd to 4th digit ratio or the anogenital distance. For these reasons, studies in animal models are sometimes more effective and outcomes can be transposed to humans. Conversely, fMRI studies are extremely useful as they allow the analysis of the functional activity of specific brain areas. In conclusion, understanding the influence of maternal exposure to androgens rather than other hormones on brain development is complicated and sometimes biased by methodological limitations.

**Audience**: It would be interesting to evaluate whether there are differences in hormonal or autonomic nervous system stress responses, specifically examining whether empathetic individuals exhibit different vegetative or hormonal responses compared to non‐empathetic individuals, regardless of gender. Moreover, stress response education, particularly for specific groups of people, such as inmates or those nearing the end of life may modify their behavior. Several studies have applied mindfulness or meditation techniques to inmates, resulting in a significant reduction in their levels of aggressiveness.^79^, ^80^

Eventually, several psychiatric disorders are characterized by emotional dysregulation, antisocial behaviors, alterations in the interpersonal relationships, and the perception of others’ emotional state, and in the empathic sphere, such as narcissistic, borderline, or antisocial personality disorders, and psychopathy.^81^, ^82^ Are there gender differences?

**AA (group 2)**: Overall, psychopathy and personality disorders are more frequent in men compared to women.^83^ This is the case of narcissistic personality disorder^84^ and antisocial personality disorder,^85^ while borderline personality disorder, which is specifically characterized by emotional dysregulation, experience of interpersonal hypersensitivity, impulsivity, and difficulty regulating emotions, including anger, has a higher prevalence in women.^86^ These clinical conditions are often occurring in comorbidity with other mental health disorders, such as alexithymia, described as a deficiency in understanding, processing, or describing self and others’ emotions,^82^ which is more frequent in men.^87^, ^88^, ^89^ Some studies support the hypothesis that psychopathy and alexithymia have different gender manifestations and traits.^87^ In psychopathy, males and females display different interpersonal attitudes, and behaviors, since men have more impulsive and violent manifestations, while females are more prone to escape, self‐harming, manipulation, and fraud attitudes. Also, gender differences in psychopathy motivation were described.^90^

Nevertheless, few studies specifically compare clinical and behavioral aspects of psychiatric disorders in males and females. Indeed, only recently studies have been provided on female psychopathy and antisocial personality disorder.^87^

**MZ (referee)**: Just a comment about mirror neurons, as they are widely discussed and are my core business as a physiologist. Mirror neurons are very interesting because, unlike nearby cortical areas where neurons deal with movements, such as specific joint movements, they deal with goals.^91^, ^92^ This is why they are multimodal and why when a human sees another human doing the same thing, it does not mean they see the same movement. That would not be possible. But they see the same goal, so they are intentional neurons. Therefore, they represent the intention and the goal one wants to achieve. Hence, the great relevance of the discovery, which was accidental but then rigorously described by its discoverers leading to many consequences in various fields of neuroscience. I would also like to say a word about autism. I was surprised that it was not deeply discussed because autism is one of the fields where these issues have more counterparts. One of the theories about the reasons for the strong prevalence of autism among males compared to females is that there are problems during brain development due to excessive exposure to male hormones or various types of environmental interferents or forms of perinatal stress that make male sex hormones, such as testosterone, more effective in brain development, hence the theory that the autistic brain is a super male brain.^83^

## 4 APORETIC CONCLUSION

**AA (group 2)**: In conclusion, empathy is a multidimensional construct and defining it may be difficult given the various perspectives and disciplines from which it can be approached (e.g., psychology, neuroscience, philosophy, and sociology). However, it can be reliably distinguished in emotional and cognitive components.

Gender‐related differences are more evident in affective empathy and their presence at a phylogenetic and ontogenetic level indicates a strong biological root. Among the biological determinants, testosterone seems to play an important role. As shown by CL (group 1), studies on human infants demonstrate that the level of empathy at various ages from birth negatively correlates with the degree of fetal testosterone exposure. Moreover, other data indicate that testosterone administration in healthy women impairs their empathic skills. However, as MC (group 2) diligently illustrated, the diversity of empathy between men and women cannot be entirely attributed to testosterone because other hormones, such as vasopressin, also act at the brain level with different effects depending on gender. The action of testosterone itself is secondary to the cortisol activity, as the dual‐hormone hypothesis supports, and female hormones also play a role in determining empathy as seen in studies in reproductive‐age women at different stages of the menstrual cycle. Additionally, structural and functional gender‐related differences in specific neural networks, namely mirror neuron system, cannot be ignored.

Nevertheless, for empathy as for all other multifactorial processes, it is necessary to take into account the role of social factors. Literature data show that maternal sensitivity, a secure attachment in infancy, and belonging to collectivist societies are some factors that seem to be associated with greater empathic skills.^3^, ^77^, ^78^ Interestingly, individual differences in empathy are related to the sexual orientation of the subject.^72^ Another social factor to consider is the gender expectation gap. Women tend to perform better in self‐assessment tests because they want to appear more empathetic to be recognized in the caregiver role attributed to them by society. In fact, in studies where women were told to complete the questionnaire because it was investigating their empathy, they performed better compared to studies where the purpose of the study was not explicitly stated.^3^, ^93^ Furthermore, when asked to fill out military aptitude tests, women tended to perform worse, probably to feel more recognized in a traditionally male‐dominated profession.^15^ Eventually, we must mention that empathy is supposed to be evolved as adaptive behavioral responses to the environment,^94^ thus historical and cultural context cannot be neglected. Indeed, the social‐role theory supports the hypothesis that socialization drives empathy and empathic sex differences, as a trait acquired and achieved from societal and cultural factors.^95^, ^96^ As an interpersonal and collective feature, empathy can be influenced by group membership, power status, moral and social similarities. Personalities may also be influenced by cultural norms adopted and ascribed to a single individual, which concur in generating gender‐role stereotypes.^96^ The identification of environmental influence on empathic traits has been described in studies conducted on identical twins, in which a socio‐cultural impact from family members on individual personality was observed.^97^ However, empathic manifestations present very early in life, with differences between the two sexes that may be attributed to biological determinants.^83^

**MZ (referee)**: Thank you for delving into so many aspects of empathy. It is evident that there is a consistent gender difference, and that testosterone has a predominant role in inducing it, although it is not the only responsible.

## Data Availability

Data sharing is not applicable to this paper as no new data were created or analyzed in this study.
